# Pathway-Centric Structure-Based Multi-Target Compound Screening for Anti-Virulence Drug Repurposing

**DOI:** 10.3390/ijms20143504

**Published:** 2019-07-17

**Authors:** Li Xie, Lei Xie

**Affiliations:** 1 Department of Computer Science, Hunter College, The City University of New York, New York, NY 10065, USA; 2 Program in Computer Science, Biochemistry & Biology, The Graduate Center, The City University of New York, New York, NY 10016, USA

**Keywords:** virtual screening, polypharmacology, phenazine biosynthesis pathway, protein–ligand docking, structure-based drug design

## Abstract

The emergence of superbugs that are resistant to last-resort antibiotics poses a serious threat to human health, and we are in a “race against time to develop new antibiotics.” New approaches are urgently needed to control drug-resistant pathogens, and to reduce the emergence of new drug-resistant microbes. Targeting bacterial virulence has emerged as an important strategy for combating drug-resistant pathogens. It has been shown that pyocyanin, which is produced by the phenazine biosynthesis pathway, plays a key role in the virulence of *Pseudomonas aeruginosa* infection, making it an attractive target for anti-infective drug discovery. In order to discover efficient therapeutics that inhibit the phenazine biosynthesis in a timely fashion, we screen 2004 clinical and pre-clinical drugs to target multiple enzymes in the phenazine biosynthesis pathway, using a novel procedure of protein–ligand docking. Our detailed analysis suggests that kinase inhibitors, notably Lifirafenib, are promising lead compounds for inhibiting aroQ, phzG, and phzS enzymes that are involved in the phenazine biosynthesis, and merit further experimental validations. In principle, inhibiting multiple targets in a pathway will be more effective and have less chance of the emergence of drug resistance than targeting a single protein. Our multi-target structure-based drug design strategy can be applied to other pathways, as well as provide a systematic approach to polypharmacological drug repositioning.

## 1. Introduction

The emergence of multi-drug resistance and extensively drug-resistant microbes to antibiotics poses a great threat to human health [1]. As described by the World Health Organization and others, we are in a “race against time to develop new antibiotics” [2]. New approaches are needed to control drug-resistant pathogens and reduce the emergence of multi-drug-resistant microbes, which are costly to treat and can lead to serious treatment failures [3]. Targeting virulence factors and pathogen–host interactions has emerged as a promising strategy in antibacterial drug discovery [4]. Such an approach places less selective pressure on bacteria to evolve new strategies for survival, and is therefore more specific to pathogens and subject to less selection for drug resistance.

In this study, we focus on repurposing FDA-approved drugs to reduce the virulence of *P. aeruginosa*. *P. aeruginosa* is an opportunistic, gram-negative bacterial pathogen that causes infections in immune-compromised hosts, burn victims, individuals in intensive care, and patients with cystic fibrosis (CF). The lungs of nearly all CF patients are chronically colonized by *P. aeruginosa*, which significantly reduces life expectancy and is the leading cause of morbidity and mortality for CF patients. The effectiveness of *P. aeruginosa* as a pathogen can be attributed to its arsenal of virulence mechanisms and its large metabolic capacity, including its ability to intrinsically resist antibiotics owing to its impermeable outer membrane, efflux capabilities, tendency to colonize surfaces in a biofilm form, and ability to acquire and maintain antibiotic plasmids [5]. Novel approaches for the treatment of *P. aeruginosa* infection are urgently needed.

From our previous study, a selective estrogen receptor modulator (SERM) raloxifene—a drug currently used in the prevention of osteoporosis and/or invasive breast cancer in post-menopausal women, as well as the treatment of gynaecomastia in men [6]—was discovered to strongly attenuate *P. aeruginosa* virulence in a *C. elegans* model of infection [7]. Raloxifene is predicted to bind *P. aeruginosa* PhzB2, which is involved in the production of the blue pigment pyocyanin produced via the phenazine biosynthesis pathway [8]. Pyocyanin is toxic to eukaryotic cells and has been shown to play a key role in infection, making it an attractive target for anti-infective drug discovery. The treatment of *P. aeruginosa* wild-type strains PA01 and PA14 with raloxifene resulted in a dose-dependent reduction in pyocyanin production in vitro. These results suggest that targeting the phenazine biosynthesis pathway may be suitable for further development as a therapeutic method for treating *P. aeruginosa* infections, and such already-approved drugs may be computationally screened and potentially repurposed as novel anti-virulence agents.

Multi-target therapy (also known as polypharmacology) [9,10] and combination therapy have been actively pursued to boost clinical efficacy and overcome acquired drug resistance in the treatment of diseases such as cancer and diabetes. Here, our goal is to identify existing drugs which could target multiple proteins on the phenazine biosynthesis pathway. Phenazines are pigmented, redox-active, heterocyclic, nitrogen-containing molecules secreted by a considerable number of bacteria, including multiple fluorescent Pseudomonas species, which enable them to act as broad specificity antibiotics as well as virulence and survival factors. Inhibitors that target this pathway will reduce the virulence of bacteria, but will not kill targeted pathogen [7]. Therefore, such inhibitors will only impose a limited growth selection pressure and are less likely to prompt drug resistance. Protein–ligand docking is commonly used to rapidly screen a library of putative drugs for their potential to inhibit protein targets. In this study, we extend protein–ligand docking to identify drug candidates that can target several proteins simultaneously. Here, we screen 2004 clinical and pre-clinical drugs to target five enzymes in the phenazine biosynthesis pathway, using a novel procedure of protein–ligand docking. Our detailed analysis suggests that kinase inhibitors, notably Lifirafenib, are promising lead compounds for inhibiting the phenzine biosynthesis, and merit further experimental validations. Our multi-target structure-based drug design strategy can be applied to other pathways, as well as provide a systematic approach to polypharmacology and drug repositioning that is actively pursed in tackling drug-resistant pathogens and other complex diseases—such as cancer and Alzheimer’s disease—by the drug discovery community.

## 2. Result

### 2.1. Target Selection and Compound Library

**Figure 1:**
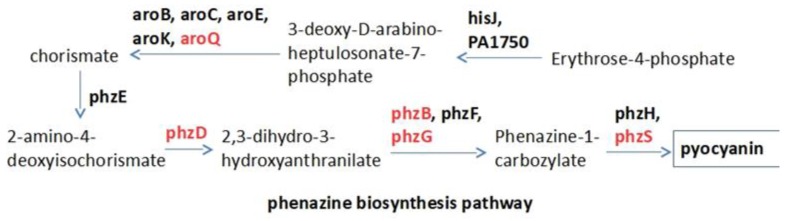
The phenazine biosynthesis pathway. Red indicates the proteins with known crystal structures.

Figure 1 shows the major enzymes that are involved in the phenazine biosynthesis pathway. Among them, there are five proteins with known PDB structures including aroQ (PDB id: 4l8l), phzD (PDB id: 1nf9), phzB (PDB id: 3ff0), phzG (PDB id: 1t9m), and phzS (PDB id: 3c96). A total of 2004 purchasable small molecules, all of which are clinical or pre-clinical drugs, are docked to these five enzymes. We do not use homology models for those enzymes which do not have solved structures, since protein–ligand docking is particularly unreliable when applied to the homology model.

### 2.2. Raw Docking Score for aroQ, phzB, phzD, phzG, phzS

PDB 4l8l is a type II dehydroquinase (aroQ) for *P. aeruginosa*. No type I dehydroquinase has been identified in the *Pseudomonas* genome using the *Pseudomonas* database, thus only type II dehydroquinase is considered for *P. aeruginosa*. Due to the absence of co-crystallized ligand in 4l8l, the active sites Arg19, Asn75, His81, Arg112, and Ser103 [11] were used to define the binding pocket for Autodock Vina screening. Raw docking scores of small molecules to aroQ showed a weak correlation with the number of their heavy atoms (Figure S1). For other proteins, the location of their co-crystallized ligand or substrate was used to define the binding pocket. Among them, the binding pockets of phzB and phzD are smaller than other proteins. Large compounds do not easily fit into their binding pockets, which can be seen from the correlation between raw docking score and the number of heavy atoms (Figure S2). phzG and phzS showed a negative correlation when the number of heavy atoms is smaller than 45, and a positive correlation when the number of heavy atoms is bigger than 45 (Figure S3). The purpose of this work is to repurpose existing drugs that can target multiple proteins in a disease-causing pathway, but not a single protein target. There are more proteins with large binding pockets (aroQ, phzG, and phzS) than those with small binding pockets (phzB and phzD) in the phenazine biosynthesis pathway. Thus, we will focus on the analysis of docking results on aroQ, phzG, and phzS.

### 2.3. Normalized Docking Score with Linear Regression Method

Generally speaking, the molecules with more atoms show better docking scores than smaller ones, because scoring functions in docking program favor enthalpic effects and mostly ignore entropic effects from unbound state to bound state. In addition, docking scores are receptor-dependent. In statistics, data normalization can transform the data with different scales to the canonical form with direct comparability. Here, in order to compare the binding scores of small molecules with different sizes and across multiple enzymes, we applied a normalization method to make the docking score more balanced between small molecules with different sizes and comparable across proteins. Thus, the ranking of the scores is more meaningful. For aroQ, phzB and phzD, a single linear regression model was used to calculate the correlation between the docking score and the number of heavy atoms for the whole set of small molecules. For phzG and phzS, two linear regression models were used for the small molecules with the number of heavy atoms smaller than 45 and larger than 45 separately. The linear regression models for each protein were shown in Supplemental Materials Figures S1–S3. From the linear fitting curve, the average docking score for molecules with a certain number of heavy atoms can be estimated and used to calculate the normalized docking score for each small molecule.

### 2.4. Docking Conformations and 2D Ligand–Protein Interactions

**Figure 2:**
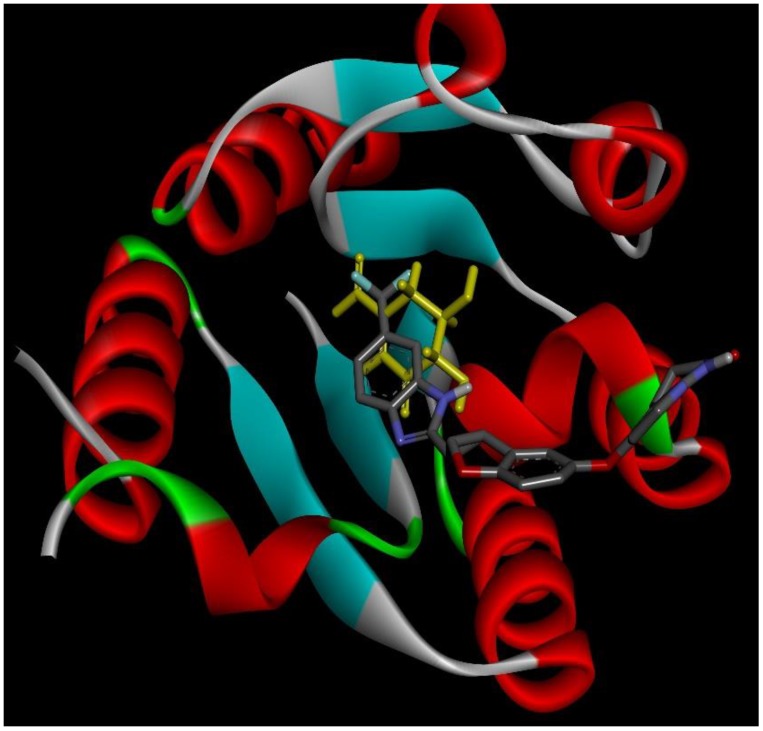
Docking conformation of Lifirafenib (gray stick) and 3DS (yellow stick) in aroQ.

**Figure 3:**
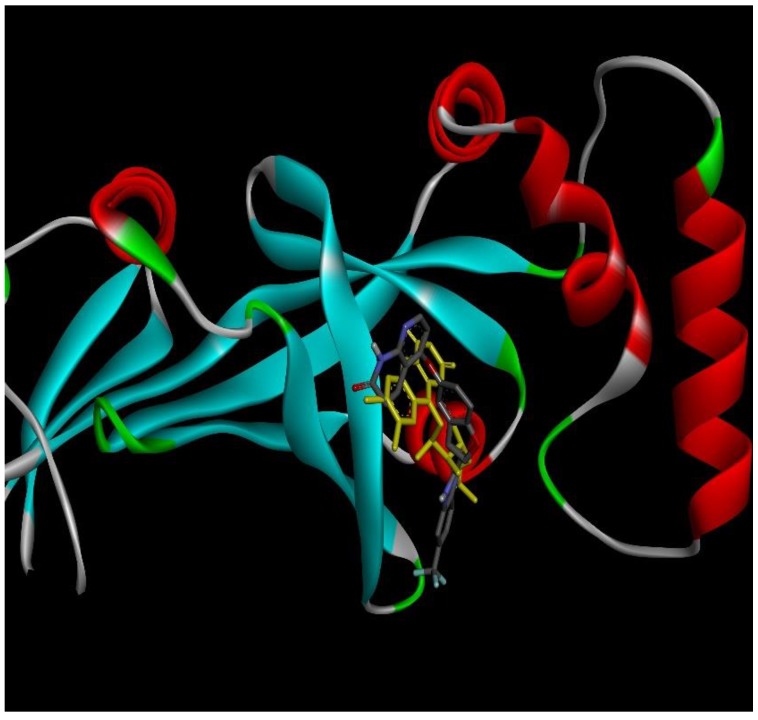
Docking conformation of Lifirafenib (gray stick) and FMN (yellow stick) in phzG.

**Figure 4:**
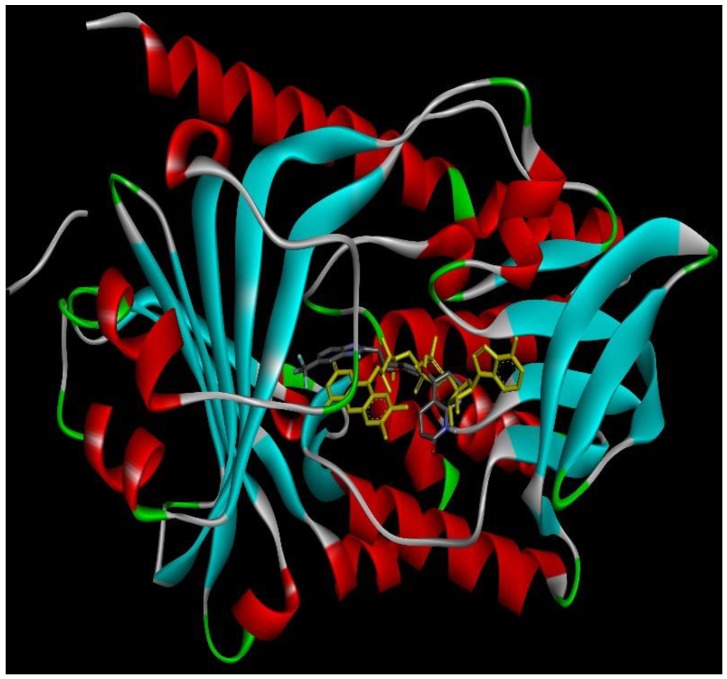
Docking conformation of Lifirafenib (gray stick) and FAD (yellow stick) in phzS.

After ranking these small molecules according to their normalized docking scores for aroQ, phzG and phzS separately, the compounds that could target all three proteins were selected, as shown in Table 1. Lifirafenib—a novel small molecule inhibitor of the RAF kinase and EGFR, which potently inhibits BRAF(V600E)-activated ERK phosphorylation and cell proliferation [12]—was predicted to inhibit aroQ, phzG and phzS. This compound is in Phase 1 of its clinical trial and has been dosed in more than 150 patients globally until now. Docking conformations of Lifirafenib were then compared with the original ligands in these proteins. The only crystal structure of aroQ from *P. aeruginosa* was 4l8l and no ligand existed in the PDB structure. In order to obtain the structure information of the substrate in aroQ, homolog structures were searched in the PDB database. PDB 3n59, the crystal structure of aroQ from M. tuberculosis, is the only one with the substrate 3DS shown in the PDB structure. Since the enzymatic sites of aroQ from *P. aeruginosa* are quite similar to its homologous enzymes [11], 3n59 was superimposed upon 4l8l to get the conformation of 3DS in the binding pocket of aroQ from *P. aeruginosa*. As shown in Figure 2, the docking conformation of Lifirafenib was compared with the superimposed 3DS conformation. For phzG (Figure 3) and phzS (Figure 4), the conformations of their co-crystallized ligands FMN and FAD can be directly compared with Lifirafenib. Lifirafenib occupied the binding pockets of aroQ, phzG and phzS in a similar way to their co-crystallized ligands.

**Figure 5:**
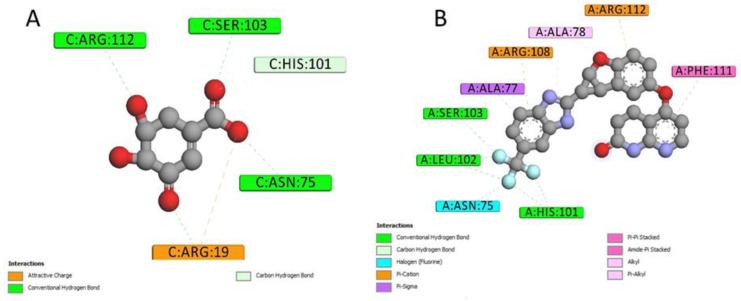
2D ligand–protein interactions for Lifirafenib and co-crystallized ligand in aroQ. (**A**) 2D interactions between 3DS and aroQ. (**B**) 2D interactions between Lifirafenib and aroQ.

**Figure 6:**
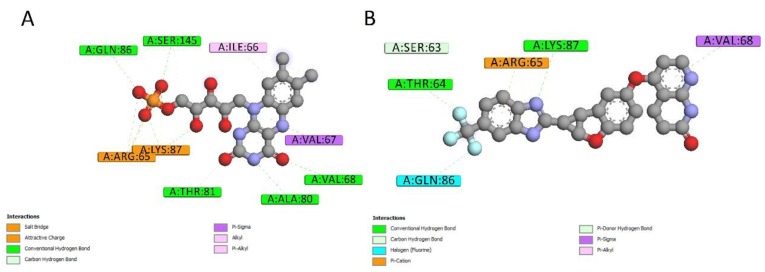
2D ligand–protein interactions for Lifirafenib and co-crystallized ligand in phzG. (**A**) 2D interactions between FMN and phzG. (**B**) 2D interactions between Lifirafenib and phzG.

**Figure 7:**
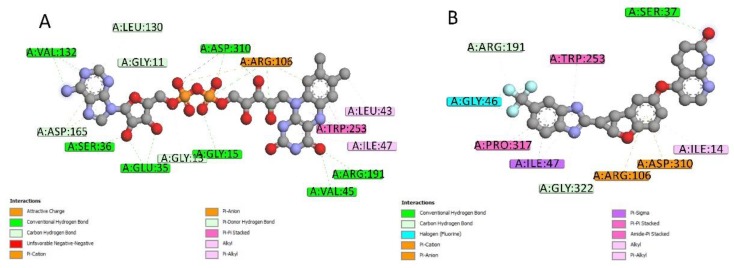
2D ligand–protein interactions for Lifirafenib and co-crystallized ligand in phzS. (**A**) 2D interactions between FAD and phzS. (**B**) 2D interactions between Lifirafenib and phzS.

2D interactions diagrams between Lifirafenib and aroQ, phzG and phzS were created using Discovery Studio Visualizer client 2019 [13]. When compared with the interactions between the proteins and their co-crystallized ligands, most of the interactions were also found between them and Lifirafenib. As shown in Figure 5, attractive charge interaction between Arg and 3DS, and hydrogen bond interactions between His101, Asn75, Ser103 and 3DS, can also be found between 4l8l and Lifirafenib—except that there are more interactions between aroQ and Lifirafenib, which makes Lifirafenib a competitor to inhibit aroQ in *P. aeruginosa*. For phzG, shown in Figure 6, several key residues—such as Arg65, Val68, Gln86, Lys87—were observed to form interactions with both co-crystallized ligand, FMN and Lifirafenib. The interactions between FAD, the co-crystallized ligand, and the residues in the binding pocket on phzS—shown in Figure 7—were also conserved when Lifirafenib bonds to phzS, including the interactions with Ile47, Arg106, Arg191, Trp253 and Asp310. Even though Lifirafenib did not form interactions with Ser36 and Val45, like FAD did, it interacted with their neighboring residues Ser37 and Gly46.

The binding scores of co-crystalized ligands on PhzG and PhzS were also calculated by Autodock Vina and compared with the raw docking scores of Lifirafenib on them. There is no complex structure for aroQ, thus only the co-crystalized ligands of PhzG and PhzS were evaluated. For PhzG, the docking score of FMN is −9.7 kcal/mol, which is slightly higher than the docking score of Lifirafenib of −11.2 kcal/mol. For PhzS, the docking scores of FAD and Lifirafenib are −10.8 and −11.6 kcal/mol, respectively. Based on the docking pose, ligand–protein interaction analysis, and the comparison of docking scores with the co-crystalized ligands, it is safe to say that Lifirafenib has high potential to inhibit aroQ, phzG and phzS on the phenazine biosynthesis pathway at the same time.

The second compound selected was Meisoindigo—a derivative of Indigo naturalis and an inhibitor of glycogen synthase kinase-3beta, GSK-3beta(ser(9)) phosphorylation in the Wnt signaling pathway [14]. The third one was JNJ-38877618, which is a Met kinase inhibitor. Interestingly, all three selected compounds are kinase inhibitors. The enrichment of kinase inhibitors here indicated that such selection was not random.

## 3. Discussion

Few existing drugs display potent anti-virulence activity in *P. aeruginosa*. Recently, it was discovered that the antibiotic azithromycin—which is not generally considered active against *P. aeruginosa*—can reduce virulence [15]. However, antibiotic resistance to azithromycin is common and has already emerged [16]. It has also been observed that azithromycin induces biofilm formation of *P. aeruginosa* [17]. Thus, the clinical use of azithromycin in controlling drug-resistant infections of *P. aeruginosa* is uncertain. Several active compounds have been discovered which reduce the virulence of *P. aeruginosa* by inhibiting quorum-sensing that regulates the production of pyocyanin virulence factors, through experimental compound screenings [18,19,20,21,22,23,24,25,26,27]. However, few of these compounds are in the preclinical or clinical stage. It may take many years to optimize the pharmacokinetics and toxicity profiles of these compounds. Raloxifene is unique as it is the first FDA-approved drug that demonstrates anti-virulence activity in vivo via directly inhibiting pyocyanin biosynthesis [7]. The kinase inhibitors that have the potential to inhibit multiple enzymes in the pyocyanin biosynthesis pathway provide new opportunities on anti-virulence drug discovery. We are carrying out in vitro and in vivo experiments to validate this prediction.

Here, we present a new approach to perform virtual compound screening against the multiple targets of the phenazine biosynthesis pathway, in order to repurpose existing drugs as efficient anti-virulence agents. Simultaneously targeting multiple enzymes will not only enhance the therapeutic effects even if the drug-target interactions are weak, but also reduce the chance of drug resistance. Our method for the multi-target structure-based drug design can be applied to other pathways or even multiple pathways. Such inhibitions are not required to be strong on a single target, but may display a high potency in inhibiting a pathway, due to their ability to bind multiple proteins. Furthermore, the compounds used here are existing drugs, so the side effects of these drugs could be controlled.

In this work, we performed structure-based virtual screening to search for small molecule compounds that can bind to known substrate sites on the target enzymes. An alternative strategy is to use blind docking to search the whole surface of the protein and to identify novel binding pockets. The searching space of a blind docking is usually huge and time consuming. The ligands could be trapped into the local minima on the energy landscape. Thus, blind docking is usually applied to proteins that have no information about the binding sites. In addition, the identified binding site from the blind docking may not be related to the enzyme activity. Here, the selected docking pockets are all known inhibitor/substrate binding sites. Because our purpose is to screen the compounds that can inhibit multiple enzymes in the phenazine biosynthesis pathway, it is reasonable to use known substrate binding sites for the docking.

We applied a consensus ranking procedure of normalized docking scores across multiple proteins to select the potential lead compounds. Normalization can help to reduce the site dependence of the docking score and make them comparable within multiple proteins. At the current stage, the effect of different chemical properties across multiple receptors on the docking score is not clear. Therefore, we used the simplest property—the number of heavy atoms—for the normalization. It could be our next step to develop an optimized normalization model to address the site-dependent issue of docking scores.

In addition to drug-resistant pathogens, many complex diseases such as cancer and Alzheimer’s disease involve multiple pathways in the disease on-set and progress. Targeting multiple proteins in a single or in multiple pathways is necessary for the development of effective therapeutics for the complex disease. The strategy presented in this study can be applied to other complex diseases, and may thus have a broad application. 

## 4. Materials and Methods

### 4.1. Drug Library

A total of 2004 small molecules provided by MedChem Express, LLC (Monmouth Junction, NJ, USA) were downloaded from Zinc15 [28]. These compounds have validated biological and pharmacological activities and many of them are FDA-approved drugs. When measured by the number of heavy atoms, their sizes vary from 5 to 72. Distributions of other physical properties with the number of heavy atoms for these compounds are shown in Figure S4, including the topological polar surface area (TPSA), Lipophilicity (LogP), Water Solubility (Ali LogS) and Pharmacokinetics (Log Kp).

### 4.2. Autodock Vina Screening

Molecular docking is widely used to estimate the binding affinity and analyze interactions between small molecules and proteins. Here, we used AutoDock Vina [29] to bring the small molecules into the active sites of the five proteins. Firstly, the PDB format of proteins and SDF format of small molecules were converted to Autodock PDBQT format. The proteins were assigned with Gasteiger Partial charges and further hydrogen atoms were added. The binding pocket for each protein was defined by its co-crystallized ligands. The center of the co-crystallized ligand was set as the center of the binding pocket. The largest distance of the ligand atoms to the center of the ligand was calculated for each x, y, and z direction to define the edge of each ligand. Twelve Angstrom of extra space was added to the ligand edge to set up the search space for the docking. Small molecules were docked to the pre-defined binding pocket. The Binding energies between receptor and the ligands were attained in terms of Kcal/mol.

### 4.3. Linear Regression

Generally speaking, the molecules with more atoms show better docking scores than smaller ones, because scoring functions in docking programs favor enthalpic effects and mostly ignore entropic effects from unbound state to bound state. In statistics, data normalization can transform the data with different scales to the canonical form with direct comparability. Here, in order to compare binding scores for small molecules with different sizes, we applied a normalization method to make the docking score more balanced between small molecules with different sizes and the ranking of the scores more meaningful. For aroQ, a single linear regression model was used to calculate the correlation between the docking score and the number of heavy atoms for the whole set of small molecules. For phzG and phzS, two linear regression models were used for the small molecules, with the number of heavy atoms smaller than 45 and larger than 45 separately.

### 4.4. Normalized Docking Score

From the linear fitting curve, the average docking score for molecules with a certain number of heavy atoms can be estimated and then the standard deviation calculated based on the distribution of docking scores and the fitted average docking score in this group. The normalized docking score NDS is calculated as a z-score:

NDS = (*S_i_* − *μ_i_*)/σ_*i*_ (1)

where *S_i_* is the raw docking score for the molecule with *i* heavy atoms, *μ_i_* is the fitted average docking score for the group of small molecules with *i* heavy atoms, and σ_*i*_ is the standard deviation calculated for this group.

### 4.5. Consensus Ranking of Small Molecules

The 2004 small molecules were ranked according to their normalized docking scores. For each protein, fifty small molecules with the lowest scores were selected and considered as the most interesting group. If a small molecule appears to be in such a group for all proteins—called a consensus ranking—this molecule could be the most promising drug to affect the whole pathway.

### 4.6. 2D Interactions between Small Molecules and Proteins

2D ligand–protein interactions were identified using discover studio, based on the docking conformations for small molecules or co-crystallized structures of original ligands.

## Figures and Tables

**Table 1:** Consensus ranking of normalized docking score on aroQ, phzG and phzS.

MCE ID	# of Heavy Atoms	Drug Name	Ranking of Z-Score			Bioactivities
			aroQ	phzG	phzS	
HY-18957	35	Lifirafenib	1	9	39	Raf Kinase and EGFR inhibitor
HY-13860	21	Meisoindigo	43	24	14	derivative of Indigo naturalis
HY-111050	28	JNJ-38877618	49	49	17	Met kinase inhibitor

## Supplementary Materials

Supplementary materials can be found at https://www.mdpi.com/1422-0067/20/14/3504/s1.
