# Supplementary figures and images for: Pathway-Centric Structure-Based Multi-Target Compound Screening for Anti-Virulence Drug Repurposing
Source: Int J Mol Sci. 2019 Jul 17;20(14):3504. doi: 10.3390/ijms20143504 (PMC6678309; doi:10.3390/ijms20143504)

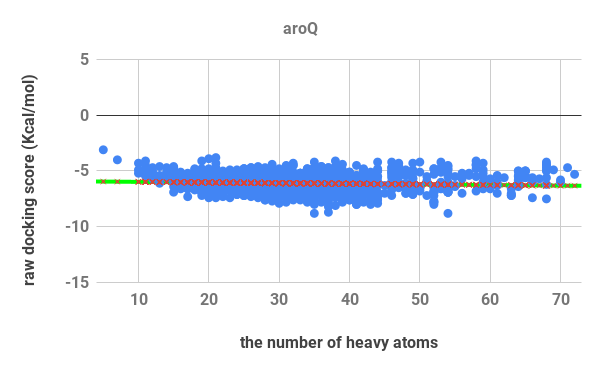

Supplement: Supplementary file 1 [file ijms-20-03504-s001.zip › Figure S1.png]

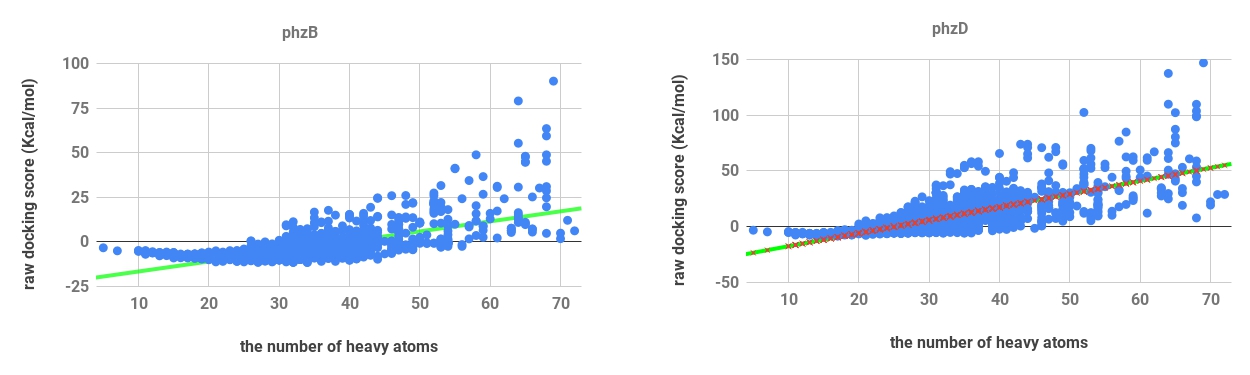

Supplement: Supplementary file 1 [file ijms-20-03504-s001.zip › Figure S2.jpg]

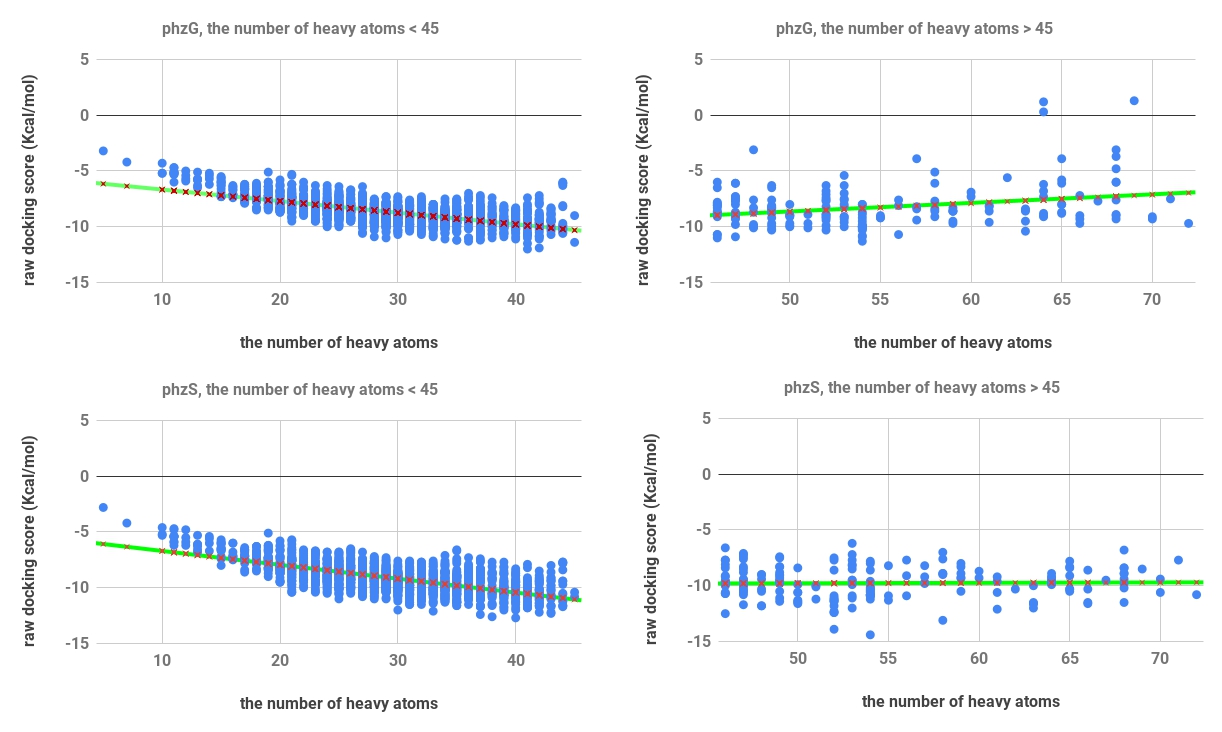

Supplement: Supplementary file 1 [file ijms-20-03504-s001.zip › Figure S3.jpg]

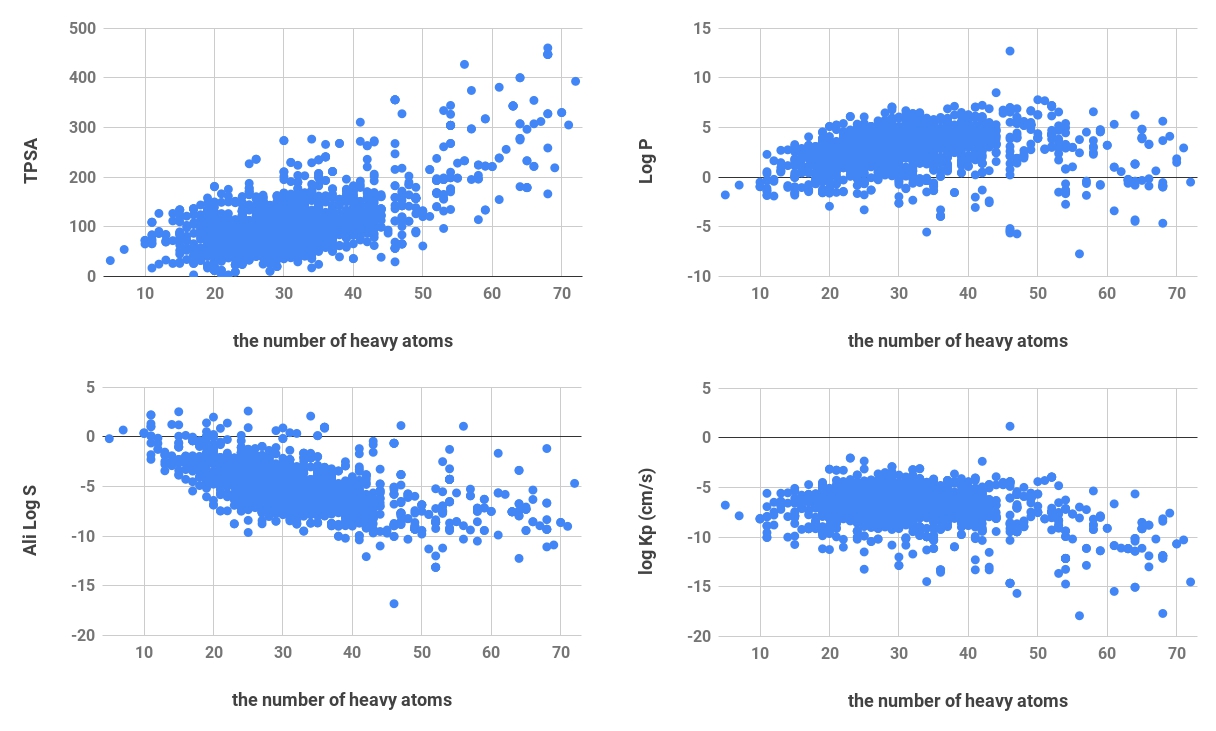

Supplement: Supplementary file 1 [file ijms-20-03504-s001.zip › Figure S4.jpg]
